# A systematic review of online interventions for mental health in low and middle income countries: a neglected field

**DOI:** 10.1017/gmh.2015.10

**Published:** 2015-07-14

**Authors:** R. Arjadi, M. H. Nauta, N. Chowdhary, C. L. H. Bockting

**Affiliations:** 1 Department of Clinical Psychology and Experimental Psychopathology, University of Groningen, Grote Kruisstraat 2/1, Groningen, The Netherlands; 2 International Medical Corps, 1313 L St. NW, Washington, DC, USA; 3 Department of Health and Clinical Psychology, Utrecht University, Heidelberglaan 1, Utrecht, The Netherlands

**Keywords:** Interventions, low-middle income countries, mental health, mental health gap, online interventions

## Abstract

**Background.:**

Low and middle income countries (LMICs) are facing an increase of the impact of mental health problems while confronted with limited resources and limited access to mental health care, known as the ‘mental health gap’. One strategy to reduce the mental health gap would be to utilize the internet to provide more widely-distributed and low cost mental health care. We undertook this systematic review to investigate the effectiveness and efficacy of online interventions in LMICs.

**Methods.:**

We systematically searched the data-bases PubMed, PsycINFO, JMIR, and additional sources. MeSH terms, Thesaurus, and free text keywords were used. We included all randomized controlled trials (RCTs) of online interventions in LMICs.

**Results.:**

We found only three articles reported results of RCTs on online interventions for mental health conditions in LMICs, but none of these interventions was compared with an active control condition. Also, the mental health conditions were diverse across the three studies.

**Conclusions.:**

There is a dearth of studies examining the effect of online interventions in LMICs, so we cannot draw a firm conclusion on its effectiveness. However, given the effectiveness of online interventions in high income countries and sharp increase of internet access in LMICs, online interventions may offer a potential to help reduce the ‘mental health gap’. More studies are urgently needed in LMICs.

## Introduction

Mental, neurological, and substance abuse (MNS) disorders are so highly prevalent in all regions around the world, that they have become major contributors to morbidity and premature mortality (WHO, 2008). Fourteen percent (14%) of the global burden of disease, measured in disability-adjusted life years, can be attributed to MNS disorders. These figures pose a challenge to make the prevention and treatment of mental disorders a public health priority in both high income countries (HICs) and low-middle income countries or LMICs (Whiteford *et al*. 2013).

In contrast to the relatively high availability of psychological treatments and mental health care professionals in HICs, it is estimated that between 76 and 85% of the people with severe mental disorders receive no treatment at all in LMICs (WHO, 2013). From the financial perspective, for instance, India only has 0.06% mental health expenditures of the total budget by the health department, while China and Indonesia have none (WHO, 2011). On the other hand, HICs like England and the Netherlands have an exclusive budget for mental health expenditures of their total health budget, by 10.8 and 10.7%, respectively (WHO, 2011). Moreover, LMICs also have limited availability of mental health services, and are often characterized by a disproportionate number between patients and mental health professionals (WHO, 2008; Eaton *et al*. 2011; Kakuma *et al*. 2011; WHO, 2013). Therefore, in general, LMICs are facing difficulties in handling mental health problems, where most people who need mental health services do not receive any, known as the ‘mental health gap’ (WHO, 2008).

The WHO launched the Mental Health Gap Action Programme (mhGAP) to scale up mental health care in LMICs and stressed the strategy of providing evidence-based interventions in non-specialized healthcare settings (WHO, 2010). Online interventions can be one promising strategy to overcome this gap, given the sharp increase of internet access of individuals in these countries and that thereby is easily accessible from various places throughout a country, and may be relatively low-cost. Online interventions have been extensively studied in HICs and several meta-analyses demonstrate that they are effective in treating psychological problems and disorders. A meta-analysis of 12 studies of computer and internet-based interventions for depression in HICs reported effect sizes ranging from 0.00 to 1.30, with a mean of 0.41 (95% CI 0.29–0.54) (Andersson & Cuijpers, 2009). Another meta-analysis of 22 studies on computerized interventions for depression and anxiety in HICs showed overall effect sizes ranging from 0.28 to1.26, with mean of 0.78 (95% CI 0.59–0.96) for depression, mean of 0.92 (95% CI 0.74–1.09) for social phobia, mean of 0.83 (95% CI 0.45–1.21) for panic disorder, and mean of 1.12 (95% CI 0.76–1.47) for generalized anxiety disorder (Andrews *et al*. 2010). Recent reports indicate that online interventions are effective in HICs, but questions have been raised whether this also holds for less developed countries (Andersson & Titov, 2014).

In countries without a proper mental health insurance system (e.g. India and Indonesia), access to mental health services is limited to people with higher socio-economic status. Online interventions, referring to standardized psychological treatments provided online, in which patients can help themselves, either independently or with the help of a therapist (Donker *et al*. 2009), have the potential to be less costly and to be more efficient (Christensen, 2010). This approach increases access to mental health care with a minimum of therapist time, allowing a larger number of patients to benefit (Rochlen *et al*. 2004; Christensen, 2010). Furthermore, online interventions may partly overcome stigma associated with having a mental illness (Rochlen *et al*. 2004). Overcoming stigma is one of the reasons why online interventions have been easily accepted in HICs (Rochlen *et al*. 2004) and have the potential to help bridge the ‘mental health gap’ in LMICs.

In the current study, we provide the results of a systematic search on randomized controlled trials (RCTs) on online interventions for mental health problems in LMICs.

## Methods

### Inclusion criteria

We included RCT empirical studies on the efficacy and effectiveness of online interventions for mental health disorders or symptoms. Studies on medical problems (e.g. hypertension and diabetes) as well as studies focused on lifestyle (e.g. smoking, obesity, exercise, and nutrition) were excluded, because their main focus was not on the mental health state of the participants.

### Literature search

We conducted literature searches according to Cochrane guidelines for systematic review (Higgins & Green, 2011) in two electronic databases, PubMed, and PsycINFO. We searched these databases using a combination of MeSH terms, Thesaurus, and free text words. We defined LMIC as a country that is categorized as low, lower-middle, and upper-middle income by the World Bank based on gross national income and gross domestic product, based on the most updated release; at the moment we did the systematic search (World Bank, 2014*a*). The screening process was conducted until 1 September 2014. We used the MeSH terms and Thesaurus for the following terms: internet, online therapy, intervention, psychotherapy, randomized controlled trial, clinical trial, pilot project, case study, and developing countries. The MeSH terms and Thesaurus terms from both engines were then combined with free text LMIC, LAMIC, LAMI countr*, LMI countr*, low income countr*, middle income countr*, low-middle income countr*, lower-middle income countr*, upper-middle income countr* and also with each country's name on the World Bank's list of LMICs. all with asterisk (*) symbol, for instance Chin* for China and Chinese. We also used freetext internet or online or web- based or electronic mail* or e-therapy or web or self-help or website or computer* or e-health or e-mental health in combination with LAMIC or LMIC or LAMI Countr* or LMI Countr* or low income countr* or middle income countr* or low-middle income countr* or lower-middle income countr*, and upper-middle income countr*. In addition, we also searched using broader terms (i.e. using online or internet and psychotherapy or intervention), due to the very small number of articles and because authors may not have given information about low-middle income nor mentioned the country name in their titles or abstracts. We also examined an e-collection of web-based and mobile interventions from the Journal of Medical Internet Research (JMIR). The reference list of included articles and previous reviews were checked. If there was insufficient information in the article about the country of the study or regarding details of the study, we sent an e-mail to the corresponding author.

## Results

**Fig. 1.**
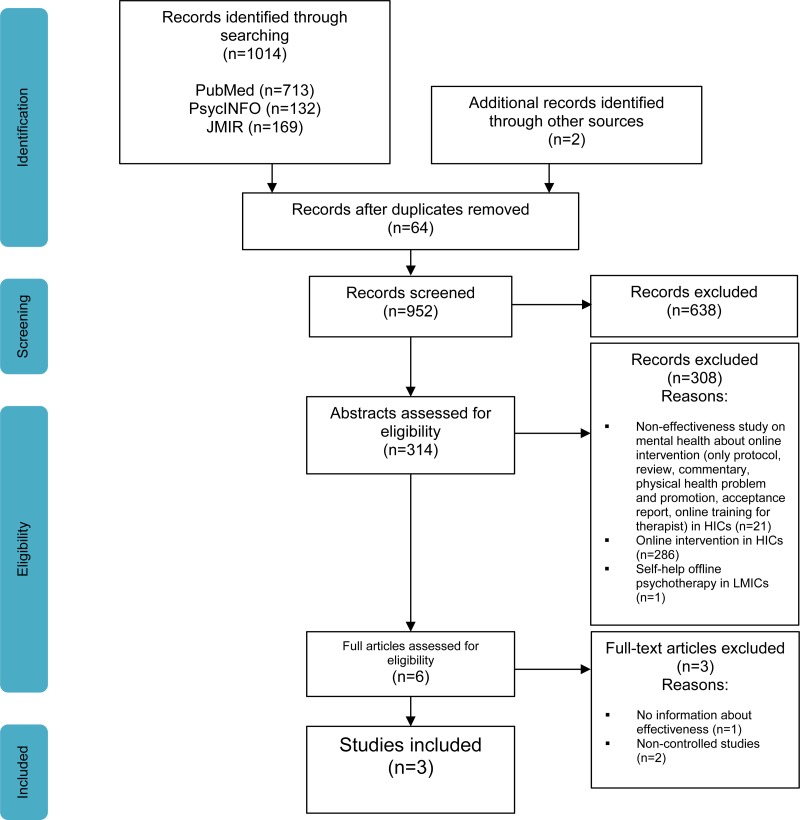
Prisma Flow Diagram. Source: Moher *et al*. (2009).

Figure 1 shows the flow diagram of the searching and screening process for the articles retrieved from all sources and search engines using Prisma Flow Diagram (Moher *et al*. 2009). After the final stage of full text eligibility assessment by R. Arjadi, C. L. H. Bockting, and M. H. Nauta, only three RCT papers were selected for inclusion in the review.

Data extraction from each study is presented in Table 1, including the description of the characteristics of each study including the effect size standardized mean differences (SMD) for the primary outcome measures.

**Table 1.** Data extraction of each study

First author and year	Recruitment	MH condition	N (originally)	Age group	Outcome measures	Intervention and number of modules/time	Supervision	Contact	Randomization	Blinding	Control group	Follow-up	Measure ment time	Dropout	Country
Wang *et al*. (2013)	Community, university and hospital counseling center	PTSD	197	18–70 year-old	PDS, SCL-D, PCC, SFI, CSE	My Trauma Recovery website (social cognitive); 6 modules; 1 month	None, except for technical difficulties in accessing the website	Phone call for recruitment and technical support	Yes	No	Wait list	3 months	Pre-test, post-test, follow-up	38.6%	China
Mogoaşe *et al*. (2013)	University student	Depressive symptoms	42	Mean: 22.87 year-old	PEQ, AMT, BDI-II, RRS, GE	Concreteness training delivered via e-mail; 7 modules; 7 days	Researcher, only to remind the participants via email if they do not finish their daily assignment	Entirely online (e-mail)	Yes	No	Wait list	None	Pre-test and post-test	2%	Romania
Su *et al*. (2011)	University student	Internet addiction	65	Mean: 22.3 year-old (18–28 year-old)	YDQ, Online hours per week, online satisfaction	Healthy online self-helping center cognitive-behavioral therapy (CBT): 4 modules; 1 day	None	Interview for screening; Feedback via website; sms for follow-up reminder	Yes	No	Wait list	None	Pre-test and post-test (mentioned as follow-up 1 month in the article)	9%	China

PTSD, post-traumatic stress disorder; PDS, post-traumatic diagnostic scale; SCL-D, symptom checklist 90-depression; PCC, post-traumatic cognitive changes; SFI, social, functioning impairment; CSE, trauma coping self-efficacy scale; PEQ, problem elaboration questionnaire; AMT, autobiographical memory test; BDI-II, beck depression inventory-II; RRS, rumination response scale; GE, global evaluation/self-drowning; YDQ, young's diagnostic questionnaire .

### Study 1 (Wang *et al*. 2013)

Study 1 was a randomized waitlist controlled trial on the effectiveness of an online intervention for participants with post-traumatic stress disorder in China. The participants were recruited from the community, the university, and hospital counseling centers. Two large parallel samples (urban and rural) samples were included, both providing an intervention group as well as a wait list. The symptom reduction in the intervention group was larger than wait list in both urban and rural samples at post-test and over a 3-month follow up. At post-test, the effect size in the rural area and urban area are 1.34 and 0.81, respectively. After 3-month follow up, the effect size in the rural area was 0.99 and in the urban area was 0.87.

### Study 2 (Mogoaşe *et al*. 2013)

This is a randomized pilot study investigating concreteness training delivered via email to reduce depressive symptoms in Romania, including undergraduate students who scored high on depression. Participants were randomly assigned to an intervention group or a waiting list. There was no significant difference between the two groups in reduction of depressive symptoms. The effect size was −0.16, indicating that intervention group have higher depression level in comparison with waiting list at post-test.

### Study 3 (Su *et al*. 2011)

This study investigated the effectiveness of online cognitive-behavior-based therapy for internet addiction in university students in China, who were assigned to four experimental conditions: laboratory environmental group (where the participants received the online intervention under laboratory conditions), natural environmental group (where the participants provided with a registration code and used the online intervention in their private places), laboratory non-interactive group (where the participants used a non-interactive system of online intervention under laboratory condition), and a wait list. In comparison with the wait list control group, all the treatment groups showed that the online treatment was more effective in reducing internet addiction. The SMD effect size was 1.68 between Laboratory and Wait list, 1.43 between Natural environment and Wait list, and 1.09 between Non-interactive and Wait list.

## Discussion

Surprisingly, only three randomized controlled studies on the effectiveness or efficacy of online psychological interventions have been conducted in LMICs, and none have included an active control condition. Even though our intention with this review was to provide meta-analytic results on the effects of online interventions in LMICs, this proved not feasible due to the scarcity of trials. This finding is especially unexpected given the high prevalence and increasing number of mental health problems worldwide. However, we do have clear evidence that online interventions are effective in HICs. Therefore it is crucial to study the effects of these online interventions in LMICs.

A prerequisite of online interventions is the availability of a good internet connection, and fortunately, internet access has improved rapidly throughout the world over the past decade. A recent survey (Pew Research Center, 2014), shows that 20% of people use the internet daily in 15 of the 24 LMICs. This is considered to be a significant number. Moreover, the number of internet users in LMICs has been increasing up to 4% each year from 5.7% in 2004 to 26.5% in 2012 (World Bank, 2014*b*).

A question might rise of why online interventions are not investigated in LMICs as extensively as in HICs. In relation to the mhGAP background, the WHO described a certain condition in LMICs, namely a widely shared idea that all mental health interventions have to be extensive and very sophisticated and can only be delivered by professionals (WHO, 2010). In this sense, it is highly likely that face-to-face interventions are also considered better than non-face-to-face interventions in LMICs. However, recent studies have demonstrated the effectiveness of using psychological interventions in non-specialized health-care settings.

## Strengths and limitations of the review

This review was conducted using robust methodology. We systematically searched through several reliable database sources for studies on online interventions in LMICs. However, we cannot answer the question whether online interventions are effective in LMICs yet due to very limited number of studies we found. Another limitation is that we only searched for peer-reviewed articles published in English. There might be more articles published in local journals using the native language of the authors.

## Implication

There is an alarming lack of RCTs in LMICs investigating the effectiveness of online interventions. Therefore, more high quality RCT studies on the efficacy and effectiveness of online interventions are urgently needed, using an active control condition as comparison to evaluate the effects in these countries. We hope that this systematic review can be an impulse to start studies on online interventions in LMICs, in order to bridge the ‘mental health gap’.
